# Frequency and outcomes of surgical and transcatheter closure of patent ductus arteriosus in preterm infants in Germany—a prospective nationwide hospital-based surveillance study

**DOI:** 10.1007/s00431-026-07073-4

**Published:** 2026-05-20

**Authors:** Kley Adelheid, Arnold Leonie, Urschitz Michael S., Müller Andreas, Singer Dominique, Gaertner Vincent D., Haas Nikolaus A., Flemmer Andreas W., Jakob André

**Affiliations:** 1 Division of Neonatology, Department of Pediatrics, Dr. von Hauner Children’s Hospital, LMU Medizin, LMU University Hospital, Ludwig-Maximilians-Universität München, Munich, Germany; 2 Department of Pediatric Cardiology and Intensive Care, LMU Medizin, LMU University Hospital, Ludwig-Maximilians-Universität München, Munich, Germany; 3 German Paediatric Surveillance Unit (GPSU), Institute of Medical Biostatistics, Epidemiology and Informatics, University Medical Centerof the, Johannes Gutenberg University Mainz , Langenbeckstraße 1, 55131 Mainz, Germany; 4 Department of Neonatology and Pediatric Intensive Care Medicine, University of Bonn, University Hospital Bonn, Bonn, Germany; 5 Division of Neonatology and Pediatric Critical Care Medicine, University Children’s Hospital, University Medical Center Hamburg-Eppendorf, Hamburg, Germany; 6 German Center of Lung Research (DZL), Comprehensive Pneumology Center Munich, Munich, Germany

**Keywords:** Preterm infants, Patent ductus arteriosus, Germany, Medical treatment, Surgical closure, Cardiac catheter/TCPC

## Abstract

Treatment strategies in patent ductus arteriosus (PDA) in preterm infants (PIs) have evolved in recent years and vary substantially between centers. While recent recommendations increasingly advocate for a more conservative approach to PDA management, transcatheter PDA closure (TCPC) has simultaneously become a well-established alternative to surgical ligation—even in very small PIs—after failure of medical treatment. Particularly in light of the ongoing conflicting perspectives in the field of optimal PDA management in PIs, our objective was to compare frequency, efficiency, and complications of surgical closure versus TCPC in a real-world setting. Between 2022 and 2023, we conducted a prospective nationwide hospital-based surveillance study including preterm infants < 32 weeks gestation and < 1500 g birthweight who underwent either surgical or transcatheter PDA closure. Standardized data on PDA treatment strategies, including medical therapy and complications, were collected by the German Paediatric Surveillance Unit (GPSU) and outcomes evaluated using the Desirability of Outcome Ranking (DOOR) method. Data from 110 infants were analyzed. Ninety-three percent (102/110) received at least one kind of medical treatment before definite mechanical closure with ibuprofen most widely used followed by paracetamol. Surgical closure was performed in 70 infants and transcatheter closure in 40 infants. Infants in the surgical group were more immature at birth (24 + 3 weeks [IQR 23 + 5–25 + 3] vs. 25 + 3 weeks [IQR 24 + 3–27 + 0], *p*  0.001), had lower birthweight (635 g vs. 733 g, *p* 0.009), and had lower weight at the time of surgery compared to catheterization (915 g vs. 1200 g, *p* < 0.001). Complications were reported in 15% (10/68) after surgical closure and in 26% (10/39) after TCPC but did not differ in the DOOR analysis.

*Conclusion*: In this nationwide study from Germany, transcatheter PDA closure was performed in approximately one-third of preterm infants, whereas surgical closure remained the predominant approach, particularly in smaller and more immature infants. The high incidence of adverse events following both methods—TCPC and surgical closure—warrants close ongoing surveillance. Substantial off-label use of paracetamol reflects evolving medical practice.

**Table:** 

**What is Known:** •* PDA in PIs remains a common clinical challenge, with a wide range of treatment strategies across NICUs.* • *New transcatheter PDA closure (TCPC) devices have recently been approved for use in preterm infants weighing > 700 g, expanding interventional options in this vulnerable population.*	
**What is New:** •* Surgical PDA closure seems to be still the preferred PDA treatment option in Germany especially in more immature infants.* •* Overall safety demonstrated no meaningful difference between TCPC and surgical PDA closure.*

**Supplementary Information:**

The online version contains supplementary material available at 10.1007/s00431-026-07073-4.

## Introduction

The number of preterm infants in Germany has been stable over the past few years with about 8–9% of all newborns born before 37 weeks of gestation. Approximately 9.000 PIs are born less than 32 weeks or below 1500 g [1]. Especially very low birthweight infants (VLBWI) < 1500 g are at high risk for comorbidities, with persistence of a ductus arteriosus (PDA) being one of them. Hemodynamically significant PDA (hsPDA) is attributed to cause diverse problems like intraventricular hemorrhage (IVH) [2] [3], bronchopulmonary dysplasia (BPD) [4], necrotizing enterocolitis (NEC), periventricular leukomalacia (PVL) and renal failure. In RCTs, there was essentially no advantage to pharmacological therapy of PDA compared to watchful waiting and there may even be disadvantages. There are currently no established criteria for PDA relevance or specific management recommendations for PIs. The decision to close a PDA depends on multiple factors and management practices vary significantly across NICUs. While pharmacological treatment with ibuprofen, indomethacin or paracetamol remains first-line, these therapies are not always effective and carry potential adverse effects [5]. Surgical ligation, traditionally used in cases of medical treatment failure, is associated with significant morbidity, including chylothorax, vocal cord or diaphragmatic paresis and long-term neurodevelopmental consequences [6, 7]. Recent evidence suggests a shift toward transcatheter PDA closure (TCPC), a less invasive alternative that may reduce the risks associated with surgery [8]. With newly approved devices enabling closure in infants > 700 g, transcatheter techniques are increasingly feasible in very small PIs. However, real-world data on the use, outcomes and complication rates of surgical versus transcatheter PDA closure remain limited, particularly in Germany and Europe.

This study aims to evaluate current management practices, including medical treatment, and determine the rates of surgical and transcatheter PDA closure in preterm infants born before 32 weeks of gestation and weighing < 1500 g in Germany, focusing on associated complications, short-term outcomes and the comparative benefits and risks of transcatheter versus surgical closure.

## Materials and methods

We performed a prospective nationwide hospital-based surveillance study over a 2-year period between 1 January 2022 and 31 December 2023 in Germany including all preterm infants born less than 32 weeks of gestation and below 1500 g with surgical or transcatheter PDA closure before discharge.

### Data sources

Data were collected in deidentified form through the German Paediatric Surveillance Unit (GPSU) with monthly reporting intervals. All pediatric hospitals were required to report the number of cases per month to a centralized clinical web portal operated by the GPSU. In a second step, a standardized online case report form—including plausibility checks—was completed. Upon survey completion and data quality checks, all data were digitally transferred to the principal investigator for analysis. This study was performed in accordance with the ethical and data protection regulations of the GPSU, which were approved by the Ethics Committee of the State Medical Association of Rhineland-Palatinate in accordance with the Declaration of Helsinki (study nr. 2020–15400).

### DOOR analysis

To allow a more detailed comparison of complications between the two groups—differentiating between severe and nonsevere events—we applied the Desirability of Outcome Ranking (DOOR) method. DOOR is an emerging analytical framework, developed to address the limitations of conventional outcome measures in comparative treatment studies [9] [10]. The DOOR approach considers benefits and harms of different strategies using, e.g., a classification from the most to the least desirable like (a) patient survival without any adverse event, (b) survival with minor adverse events, (c) survival with major adverse events, to (d) death in order to describe outcomes in a more granular manner [11, 12]. The DOOR probability represents the possibility that a randomly selected preterm infant will have a better DOOR rank if assigned to surgery compared to their DOOR rank had they been assigned to cardiac catheter. A DOOR probability near 50% indicates there is no difference in DOOR distributions by treatment group. In our study, we used the DOOR with following ranks and components: ranking: 1—alive and no event, 2—alive and nonsevere event, 3—alive and severe event; components (more than one possible in one patient): 1—nonsevere complication, 2—severe complication. We used the “DOOR Analyses: Standard Edition” online R shiny application (https://methods.bsc.gwu.edu/). All tests were considered exploratory to gain additional insight into the outcome of the different treatment pathways. In our series, severe complications were defined as, e.g., intraventricular hemorrhage > stage 2, renal thrombosis. Nonsevere complications were defined as, e.g., blood transfusion, vocal cord paresis, pneumothorax, bleeding, and remaining PDA shunt.

### Statistical analysis

Statistical analyses were performed using R statistics version 4.4.2. Data are presented as median and interquartile ranges (Q1, Q3). Missing data, if present, are reported in Table 1 and in the results. Statistical comparisons between groups were performed using Wilcoxon rank sum test, chi-square tests, or Fisher’s exact test. If data was missing, available case analysis was performed. All tests were considered exploratory to gain additional insight into the outcome of the different treatment pathways. All results with *p* < 0.05 were considered statistically significant.

**Table 1:** Patient characteristics and PDA variables

Characteristic	*N*	Overall *N* = 110^a^	Treatment group		*p* value^b^
			Surgery *N* = 70^a^	Transcatheter closure *N* = 40^a^	
Gestational age (weeks + day)	110	24 + 6 (23 + 6, 25 + 5)	24 + 3 (23 + 5, 25 + 3)	25 + 3 (24 + 3, 27 + 0)	0.001
Birthweight (grams)	110	660 (550, 770)	635 (525, 720)	733 (585, 880)	0.009
Sex (male)	110	57 (52%)	37 (53%)	20 (50%)	0.8
PDA diameter (mm)	90	2.50 (2.00, 3.00)	2.30 (2.00, 2.80)	2.50 (2.00, 3.00)	0.4
Time of diagnosis (day of life)	107	6 (4, 12)	6 (4, 11)	6 (3, 17)	0.9
Weight at closure (grams)	100	995 (843, 1290)	915 (783, 1110)	1200 (915, 1950)	< 0.001
Age at closure (day of life)	108	29 (22, 43)	28 (22, 39)	30 (21, 66)	0.2
Invasive ventilation before closure	110	74 (67%)	57 (81%)	17 (43%)	< 0.001
PDA closed at discharge	96	96 (100%)	60 (100%)	36 (100%)	> 0.9

^a^Median (Q1, Q3); *n* (%)

^b^Wilcoxon rank sum test; Pearson’s chi-squared test; Fisher’s exact test

## Results

### Study population

**Fig. 1:**
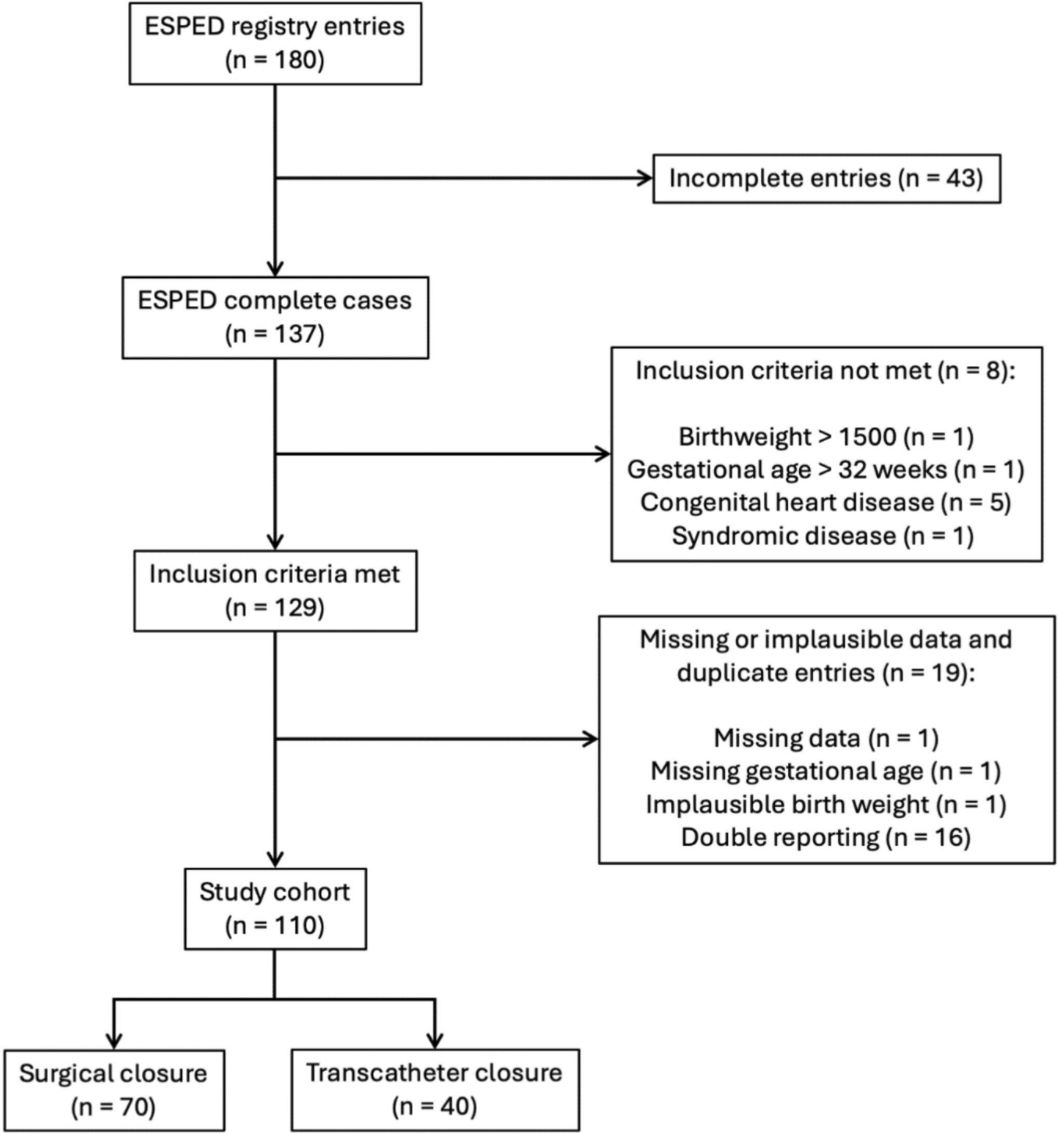
Flowchart—eligible patients and patients included for analysis after exclusions

Between January 2022 and December 2023, a total of 180 cases were reported across 65 hospitals. In 2022, 86 patients were reported to the GPSU with a questionnaire response rate of 84.2% (*n* = 74). In 2023, 93 patients were reported with a response rate of 67.7% (*n* = 63), resulting in a total of 137 completed questionnaires from 55 distinct hospitals. After exclusion of 27 infants because they either did not meet the inclusion criteria, had missing or implausible data or were reported twice, 110 infants were included to the final analysis (*n* = 70 underwent surgical closure = surgery group and *n* = 40 received transcatheter closure = TCPC group) (see Fig. 1).

#### Patient cohort

Neonatal data and post-delivery management.

The most common causes of prematurity in our sample of 106 mothers with a median age of 32 years (IQR 28, 36) were premature rupture of membranes (PROM), amniotic infection syndrome (AIS), preeclampsia/HELLP syndrome, and placental insufficiency/fetal growth restriction (FGR). Basic neonatal characteristics, including completion of antenatal corticosteroid therapy, APGAR scores, mode of delivery, and the type of surfactant application after birth, did not differ significantly between the surgery and TCPC groups (see Table 3). The overall median age of gestation of the 110 PIs was 24 + 6 weeks (IQR 23 + 6, 25 + 5); the median birthweight was 660 g (IQR 550, 770). Fifty-two percent (*n* = 57) were male PIs and 48% (*n* = 53) female. Additional demographic data are presented in Table 3 in the appendix. Ninety-nine percent of the primary care hospitals were Level III NICUs, representing the highest level of neonatal care in Germany (see Table 1).

PDA characteristics.

In 80% (*n* = 88) of the PIs, PDA was detected by routine echocardiography; 55% (*n* = 60) presented with respiratory failure, 13% (*n* = 14) with IVH ≥ 2°, 9% (*n* = 10) with heart murmur, 8% (*n* = 9) with signs of infection, and 6% (*n* = 7) with renal insufficiency. The median time of diagnosis of relevant PDA was equal in both groups at day 6. The median size of PDA diameter at time of diagnosis was not different with 2.5 mm (2.3 mm (IQR 2.0, 2.8) in the surgery group vs. 2.5 mm (IQR 2.0, 3.0) in the catheter group; *p* 0.4, *n* = 20 missing). In 63% (69/110) diastolic zero or reverse flow in the celiac artery was reported (*n* = 32 missing/unknown). Diastolic zero or reverse flow in the anterior cerebral artery was found in 50% of all PIs (55/110) (*n* = 35 missing/unknown).

#### Procedural characteristics and peri-interventional clinical status

Sixty-four percent (*n* = 70) underwent surgical closure, while 36% (*n* = 40) received transcatheter closure. Compared with PIs in the catheter group, those in the surgical group had a significantly lower median birthweight (635 g vs. 733 g, *p* 0.009) and lower gestational age at birth (24 + 3 weeks vs. 25 + 3 weeks, *p* 0.001). The median age at intervention was 29 days, with no difference between both groups (28 days [IQR 22, 39] surgery vs. 30 days [IQR 21, 66] TCPC). The median weight at the intervention was significantly lower in the surgical group (915 g [IQR 738, 1110] vs. 1200 g [915, 1950], *p* < 0.001). Furthermore, a significantly higher proportion of PIs in the surgical group required invasive ventilation before the procedure (81% [57/70] vs. 43% [17/40], *p* < 0.001) (see Table 2).

**Table 2:** DOOR distribution by treatment group (surgery vs. cardiac catheterization) with DOOR rank (alive with no event, alive with nonsevere event, or alive with severe event) and DOOR components (nonsevere or severe event) distribution

DOOR distribution	Treatment group			
	Surgery	Cardiac catherization	Gained loss (per 1000)	
	*N* (%)	*N* (%)	Per category	Cumulative
DOOR rank				
Alive and no event	57 (86.4)	31 (79.5)	− 69	− 69
Alive and nonsevere event	6 (9.1)	3 (7.7)	− 14	− 83
Alive and severe event	3 (4.5)	5 (12.8)	83	0
DOOR components				
Nonsevere event	6 (9.1)	5 (12.8)	37.3	
Severe event	3 (4.5)	5 (12.8)	82.8	

Surgery was performed in 74% (52/70) of cases in the NICU and 83% (57/69) of procedures by a pediatric cardiac surgeon (ligation in 33% and clipping in 66%). Among infants undergoing TCPC, the procedure was performed in the NICU in 44% (17/39) and in the catheterization laboratory in 46% (18/39). TCPC was performed in 84% (32/38) by a pediatric cardiologist. A Piccolo Occluder was used in 62% (21/34), a vascular plug in 21% (7/34), and a coil in 9% (3/43) of the cases; in 6 cases, there was no information about the device.

#### Pre-interventional pharmacologic treatment and supportive management

Ninety-three percent of PIs (102/110) received at least one form of medical PDA therapy, with similar rates in both groups (surgery 94% [66/70]; catheter 90% [36/40]). In contrast, patients in the surgical group received a significantly higher number of multiple pharmacological treatments as part of closure attempts (61% (40/66) vs. 36% (13/36), *p* 0.018). Approximately 8% (8/98) received both, prophylactic—initiated before showing signs or symptoms—and therapeutic treatment—initiated after symptom onset (6 surgical, 2 catheter). Four PIs in each group underwent primary mechanical closure without prior medical therapy. Ibuprofen was used in 89% (91/102) of treated PIs (*n* = 61 surgery; *n* = 30 catheter group), paracetamol in 46% (47/102) (*n* = 33 surgery; *n* = 14 catheter group), and indomethacin in 25% (25/102) (*n* = 20 surgery; *n* = 5 catheter group), with frequent combination therapy; see Supplementary Fig. 1. Approximately 75% of medical treatments were administered intravenously only. In 56% (59/106) of the PIs, fluid restriction with an average amount of fluid intake of 150 mL/kg/day in the surgery and 130 mL/kg/day in the catheter group was reported, showing no significant difference.

#### Procedural complications

Complications were reported in 15% (10/68) after surgical closure and in 26% (10/39) in the catheter group. The major complications in the surgical group were blood transfusion (3), vocal cord paresis (1), pneumothorax (1), bleeding (1), and remaining PDA shunt (1). In the catheter group, major complications were loss of pulse of the limb (2), stenosis of the left pulmonary artery (2), device dislocation (1), hematoma (1), thrombosis (1), and blood transfusion (1). In two cases, surgical closure of PDA was necessary after failure of transcatheter closure.

**Fig. 2:**
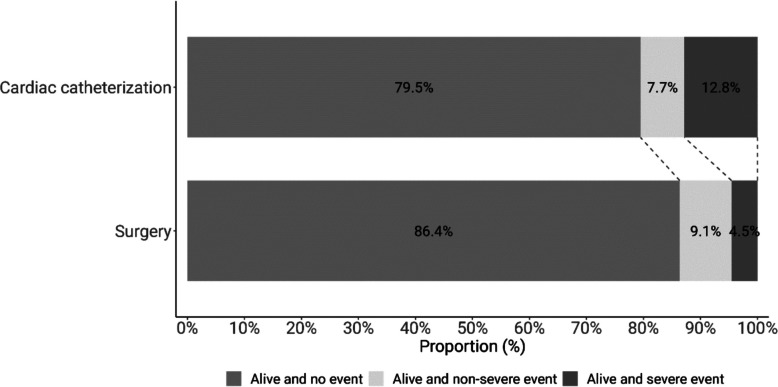
Distribution of the DOOR ranks of a cohort of preterm infants treated for persistence of a ductus arteriosus grouped by type of intervention (cardiac catheterization or surgical closure)

**Fig. 3:**
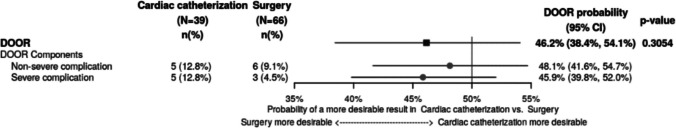
Forest plot summary of the estimated DOOR probabilities for the DOOR outcome and components within the cardiac catheterization group and the surgery group of a cohort of preterm infants treated for persistence of a ductus arteriosus. The DOOR components are based on the sequential dichotomization of the outcome (i.e., nonsevere versus severe outcome)

To compare complications of both treatment modalities, we applied the DOOR methodology, which evaluates the probability that one intervention yields a more desirable global outcome [11, 12]. In this study, the most desirable outcome was defined as no or nonsevere complication. In general, TCPC did not differ significantly from surgery regarding severe or nonsevere complications. The probability of a more favorable global outcome was 53.8% for surgery (95% CI 45.9–61.5; *p* = 0.3), indicating no meaningful advantage of either approach (see Table 2). The likelihood of achieving rank 1 (no complications) relative to all other ranks was similarly comparable between both groups (46.6%, 95% CI 38.9–54.4%). The full DOOR distribution and forest plot are shown in Figs. 2 and 3.

#### General outcome data

Ten infants died, nine in the surgical and one in the catheter group, because of complications typically associated to preterm birth like sepsis, respiratory or multiorgan failure likely unrelated to the surgical or transcatheter PDA closure. Ninety-eight of the PIs survived until discharge (*n* = 2 missing). In all of the 96 PIs, the PDA was successfully closed before discharge.

We also collected general outcome data of prematurity like NEC, BPD, and ROP. At the time of discharge, 25% (23/92) of the surviving PIs of both groups suffered from ROP stadium ≥ 3; 30% (29/96) were affected with moderate and 25% (24/96) with severe BPD. IVH ≥ 2° was reported in 27% (29/106 and NEC in 7% (7/105), whereby 6 cases of NEC occurred in the surgical group. As expected, neither of these outcome data differed statistically significantly between both groups and are more likely attributed to prematurity than to mechanical PDA closure (Table 3).

## Discussion

Although transcatheter closure of PDA in PIs has been increasingly adopted in recent years, it still must prove against surgical PDA closure, which has a long-standing role as the standard treatment following failed medical therapy. Therefore, the aim of this study was to evaluate the frequency of surgical versus transcatheter PDA closure in PIs in Germany, with particular emphasis on patient characteristics, procedure-related outcomes and complications.

Despite an international trend toward expectant and conservative management of PDA in PIs [13], a total of 180 cases undergoing either surgical or transcatheter PDA closure were reported over a 2-year period. Of these, 110 PIs were eligible for analysis, with 70 PIs receiving surgical closure and 40 PIs TCPC. Infants in the surgical group had a significantly lower gestational age and weight at birth such as lower body weight at the time of closure. They also required substantially more invasive ventilation prior to definitive PDA closure (81% vs. 43%). These findings suggest that more immature and clinically unstable infants were preferentially treated surgically. This likely reflects both clinical caution and feasibility considerations, as TCPC remains technically more challenging in smaller and sicker infants. Surgical ligation has been well established and widely accepted in German NICUs for decades, whereas TCPC in PI is a relatively recent procedure and may still be viewed with skepticism among neonatologists in some centers, particularly for very small PIs [14]. Moreover, dedicated neonatal devices for TCPC have only recently been approved [15] and experience in this dedicated population remains limited and unevenly distributed across German pediatric hospitals.

In contrast, data from the USA demonstrate a clear shift toward transcatheter PDA closure. Leahy et al. reported similar proportions of surgical and transcatheter closures in 2019 but observed a steady increase in TCPC between 2018 and 2022, with TCPC surpassing surgical ligation for the first time in 2021 [16]. Similar trends were reported by Weems et al. in a large multicenter cohort, in which TCPC became the dominant method of definitive PDA closure after 2020 [17]. Shah et al. also demonstrated a decreasing overall rate of procedural PDA closure accompanied by an increasing proportion of TCPC procedures [18]. In contrast, our data from 2022 to 2023 still show a higher proportion of surgical closures (64%). This discrepancy likely reflects the longer availability of TCPC, greater operator experience, and broader institutional adoption in the USA especially in small neonates. However, in this context, as a minor but important point, the Amplatzer Piccolo Occluder received FDA approval in the USA in January 2019 for use in infants weighing ≥ 700 g [15], whereas in Europe it has been available since 2011 under the CE mark (initially as the Amplatzer Duct Occluder II Additional Sizes), approved for larger infants, initially ≥ 6 kg [19, 20]. Substantial procedural expertise should already exist, although possibly not in infants with body weights as low as 700 g. In this context, closer interdisciplinary collaboration and clinical exchange between neonatologists and pediatric cardiologists would be highly desirable to optimize patient selection and procedural decision-making.

The median postnatal age at definitive PDA closure was 29 days and did not differ between groups. This finding may be explained by the lower gestational age at birth in the surgical group, resulting in similar postnatal timing despite different maturational stages. Our results are consistent with those of Georgiev et al., who reported a mean age of 26 days at TCPC in extremely preterm infants [21], but in contrast with other studies demonstrating later closure in TCPC compared with surgical ligation [18, 22]. These differences likely reflect evolving treatment strategies and a general shift away from early prophylactic intervention toward delayed closure following unsuccessful medical management [13, 23, 24].

Pharmacological therapy remains a cornerstone of PDA management. Almost all infants in our cohort received at least one course of medical therapy prior to definitive closure, a rate comparable to that reported by Leahy et al. [16], but higher than in other cohorts with only 20% having received medical treatment for PDA closure [17]. Although intravenous ibuprofen is currently the only approved pharmacological agent for PDA closure in small PIs, paracetamol was used in nearly half of the infants in our study. This widespread off-label use likely reflects emerging evidence suggesting comparable or superior efficacy and a favorable side-effect profile, although data on safety and long-term outcome remain limited. Katsaras et al. could show similar closure rates of PDA of all three agents in their meta-analysis, but paracetamol caused less adverse events [25]. It may be even better to completely avoid pharmacological therapy, as recent studies have shown that there was no disadvantage of watchful waiting compared to pharmacological therapy of PDA in PIs [13]. There was a high rate of multiple medications in our cohort, especially in the surgical group, which reflects the complexity of PDA and its treatment strategy. This was also shown by Mitra et al., who found a big variety of treatment modalities in their meta-analysis of 4802 infants with PDA [5].

Complications were present in both groups, particularly among infants weighing < 1000 g, and did not differ between groups. Overall, the DOOR analysis demonstrated a slightly lower probability of both severe and nonsevere complications in the surgical group, but this difference did not reach statistical significance. Considering that infants in the surgical group were more immature and weighed less and were thus likely more prone to adverse events, this is surprising and may indicate an improved outcome using surgery. However, this has to be evaluated in prospective clinical trials, powered to detect important differences. Tabb et al. reported comparable complication rates between surgical ligation and transcatheter PDA closure in preterm infants, with an overall complication rate of 20.5% (27.8% after surgical ligation vs. 17.1% after transcatheter closure). In contrast to our study, procedural complications included cardiopulmonary resuscitation and there was a higher rate of infants with catheter closure compared to our data [26]. While we do not have long-term follow-up data for our cohort, it is important to note that Kaluarachchi et al. demonstrated a significantly higher number of infants with a BSID-III cognitive composite score < 85 in their analysis of preterm infants < 27 weeks undergoing catheter closure of PDA compared to surgical ligation [27]. This is an important fact, which has to be addressed in decision-making, and further randomized studies regarding the long-term outcome after mechanical closure of PDA in PIs are needed.

Both methods are not without any harm and our data highlight the need of experienced teams in relation to both interventions. Nevertheless, complications associated with TCPC, even among very experienced teams, cannot be completely avoided. Philip et al. reported two cases of left pulmonary artery stenosis requiring device retrieval in infants ≤ 1000 g [28] and Georgiev et al. described two severe complications—one aortic coarctation and one right pulmonary artery obstruction due to device dislocation—among 58 patients [21]. Major complications following TCPC reported in our series included limb ischemia, left pulmonary artery stenosis, and device dislocation, consistent with the previously published reports. Two infants in the transcatheter group required subsequent surgical PDA closure after unsuccessful TCPC, indicating a low procedural failure rate. This finding is also consistent with previous reports, including the study by Méot et al., who analyzed 130 preterm infants referred for TCPC and observed 14 aborted procedures, with five infants ultimately requiring surgical closure. Notably, they also reported early mechanically induced spontaneous PDA closure in seven infants following unsuccessful catheterization, an observation that warrants consideration [29].

A further important consideration is the treatment location. Surgical PDA closure was predominantly performed bedside in the NICU, whereas nearly half of the TCPC procedures required transport to the catheterization laboratory. Transport of extremely preterm infants carries inherent risks and remains a relevant limitation of TCPC in many centers [30]. While bedside TCPC has been described, experience remains limited [21]. Until more data are available, structured referral pathways, safe transport protocols, and experienced multidisciplinary teams are essential [22].

## Strengths and weaknesses

The major strength of this study is its prospective hospital-based design using an established nationwide surveillance system, allowing inclusion of an unrestricted number of PIs undergoing interventional PDA closure. However, several limitations must be acknowledged. Despite active surveillance with monthly requests, reporting of cases was voluntary, likely leading to underestimation of procedural closure rates, particularly given the low questionnaire response rate in 2023. According to data protection regulations, respective case anonymization precluded center-specific analyses. Recall was not possible and detailed data on pharmacological treatment regimens, indications for closure, and long-term outcomes were unavailable. Additionally, it was not possible to retrieve or complete missing data. Consequently, a more comprehensive analysis of PDA characteristics and their hemodynamic impact could not be performed, and the rationale for choosing surgical versus transcatheter intervention was not captured in the dataset. In the absence of such data and given that no national guidelines currently provide definitive recommendations favoring one approach over the other, we assume that the choice of treatment modality was largely influenced by institutional factors, including site-specific expertise, local practice patterns, and availability of transcatheter techniques. Finally, due to the lack of national incidence data on hemodynamically significant PDA, temporal trends could not be assessed.

## Conclusion

Between 2022 and 2023, 110 preterm infants weighing < 1500 g and born before 32 weeks of gestation underwent definitive PDA closure in Germany, with 36% treated by transcatheter techniques. Surgical closure remained the predominant approach, particularly in smaller and more immature infants. Both surgical and transcatheter PDA closure were associated with substantial complication rates, underscoring the need for careful patient selection and experienced multidisciplinary care. The high use of paracetamol for medical PDA treatment highlights considerable variability in clinical practice and the frequent use of off-label therapies.

Further prospective studies, including long-term outcome data, are urgently needed to better define the optimal management strategy for PDA in preterm infants. Until such data are available, each NICU should adopt evidence-based local guidelines and employ the treatment modality with the greatest institutional expertise and safety profile.

## Supplementary Information

Below is the link to the electronic supplementary material.

ESM 1 Table 3: Additional demographic and outcome data. (DOCX 43.3 KB)

ESM 2 Supplementary Fig. 1: Rate of preterm infants in (%) of the surgical or catheterization group receiving pharmacotherapy with indomethacin, paracetamol, or ibuprofen (multiple therapy possible) with the total number of patients of each group beside the pillars. (PNG 25.0 KB)

## Data Availability

All data supporting the findings of this study are available within the paper and its Supplementary Information. Microsatellite primer sequences are provided in Supplementary Table 3.

## Abbreviations

ADD: Attention-deficit disorder
AIS: Ammniotic infection syndrome
BPD: Bronchopulmonary dysplasia
BSID: Bayley Scales of Infant Development
BW: Birthweight
DOOR: Desirability of Outcome Ranking
e.g.: For example
FGR: Fetal growth restriction
GA: Gestational age
GPSU: German Paediatric Surveillance Unit
hsPDA: Hemodynamically significant patent ductus arteriosus
IVH: Intraventricular hemorrhage
IV: Intravenous
INSURE: Intubation surfactant-application extubation
LISA: Less invasive surfactant application
NEC: Necrotizing enterocolitis
NICU: Neonatal intensive care unit
PDA: Patent ductus arteriosus
PI(s): Preterm infant(s)
PMA: Postmenstrual age
PROM: Premature rupture of membranes
PVL: Periventricular leukomalacia
SL: Surgical ligation
TCPC: Transcatheter PDA closure
VLBWI: Very low birth weight infants
