# Supplementary material for: Frequency and outcomes of surgical and transcatheter closure of patent ductus arteriosus in preterm infants in Germany—a prospective nationwide hospital-based surveillance study
Source: Eur J Pediatr. 2026 May 20;185(6):416. doi: 10.1007/s00431-026-07073-4 (PMC13186873; doi:10.1007/s00431-026-07073-4)
Supplement: Supplementary file 1 — Table 3: Additional demographic and outcome data. (DOCX 43.3 KB) [file 431_2026_7073_MOESM1_ESM.docx]

**Supplements**

Table 3: Additional demographic and outcome data.

**Additional Patients characteristics**

| **Characteristic** | **Overall** N = 110*^1^* | **Treatment group** | | **p-value***^2^* |
| --- | --- | --- | --- | --- |
|  |  | **Surgery** N = 70*^1^* | **Cardiac catheterization** N = 40*^1^* |  |
| Birth clinic: |  |  |  | >0.9 |
| Neonatology Level 1 | 107 (99%) | 69 (99%) | 38 (100%) |  |
| Neonatology Level 2 | 0 (0%) | 0 (0%) | 0 (0%) |  |
| Neonatology Level 3 | 1 (0.9%) | 1 (1.4%) | 0 (0%) |  |
| Missing | 2 | 0 | 2 |  |
| Mode of delivery: |  |  |  | 0.3 |
| Caesarean section | 90 (82%) | 55 (79%) | 35 (88%) |  |
| Vaginal birth | 20 (18%) | 15 (21%) | 5 (13%) |  |
| Apgar Score 5 min.: |  |  |  | 0.2 |
| 0-3 | 7 (6.5%) | 2 (2.9%) | 5 (13%) |  |
| 4-6 | 36 (34%) | 23 (34%) | 13 (33%) |  |
| 7-10 | 64 (60%) | 43 (63%) | 21 (54%) |  |
| Missing | 3 | 2 | 1 |  |
| Apgar Score 10 min.: |  |  |  | 0.7 |
| 0-3 | 0 (0%) | 0 (0%) | 0 (0%) |  |
| 4-6 | 9 (8.5%) | 5 (7.5%) | 4 (10%) |  |
| 7-10 | 97 (92%) | 62 (93%) | 35 (90%) |  |
| Missing | 4 | 3 | 1 |  |
| Complete course of antenatal corticosteroids | 105 | 69 (66%) | 47 (70%) | 22 (58%) |
| Surfactant application | 106 (98%) | 70 (100%) | 36 (95%) | 0.12 |
| Missing | 2 | 0 | 2 |  |
| Type of surfactant application: |  |  |  | 0.8 |
| LISA | 44 (42%) | 28 (40%) | 16 (44%) |  |
| INSURE | 6 (5.7%) | 5 (7.1%) | 1 (2.8%) |  |
| Intubation and ventilation | 56 (53%) | 37 (53%) | 19 (53%) |  |
| Missing | 4 | 0 | 4 |  |
| Arterial flow pattern celiac artery: | 110 |  |  |  |
| normal | 9 (8.2%) | 9 (13%) | 0 (0%) |  |
| zero/negative diastolic flow | 69 (63%) | 42 (60%) | 27 (68%) |  |
| unknown/missing | 32 (29%) | 19 (27%) | 13 (33%) |  |
| Arterial flow pattern A. cerebri anterior: | 110 |  |  |  |
| normal | 20 (18%) | 17 (24%) | 3 (7.5%) |  |
| zero/negative diastolic flow | 55 (50%) | 39 (56%) | 16 (40%) |  |
| unknown/missing | 35 (32%) | 14 (20%) | 21 (53%) |  |
| *^1^* Median (Q1, Q3); n (%) | | | | |
| *^2^* Wilcoxon rank sum test; Fisher’s exact test; Pearson’s Chi-squared test | | | | |

**Medication**

| **Characteristic** | **Overall** N = 110*^1^* | **Treatment group** | | **p-value***^2^* |
| --- | --- | --- | --- | --- |
|  |  | **Surgery** N = 70*^1^* | **Cardiac catheterization** N = 40*^1^* |  |
| Patients receiving medical treatment | 102 (93%) | 66 (94%) | 36 (90%) | 0.5 |
| Fluid restriction | 59 (56%) | 42 (60%) | 17 (47%) | 0.2 |
| Amount of fluid restriction | 140 (130, 150) | 150 (130, 155) | 130 (130, 150) | 0.13 |
| Timepoint of medical treatment: |  |  |  | 0.6 |
| Only prophylactic | 2 (2.2%) | 2 (3.4%) | 0 (0%) |  |
| Only therapeutic | 80 (89%) | 51 (86%) | 29 (94%) |  |
| Both | 8 (8.9%) | 6 (10%) | 2 (6.5%) |  |
| Missing | 12 | 7 | 5 |  |
| Kind of medication: |  |  |  |  |
| Indometacin | 25 (25%) | 20 (30%) | 5 (14%) | 0.066 |
| Acetaminophen | 47 (46%) | 33 (50%) | 14 (39%) | 0.3 |
| Ibuprofen | 91 (89%) | 61 (92%) | 30 (83%) | 0.2 |
| Application of medical treatment: |  |  |  | >0.9 |
| Only i.v. | 61 (73%) | 41 (73%) | 20 (71%) |  |
| Only p.o. | 15 (18%) | 10 (18%) | 5 (18%) |  |
| Both | 8 (9.5%) | 5 (8.9%) | 3 (11%) |  |
| Missing | 18 | 10 | 8 |  |
| Number of medications: |  |  |  | **0.018** |
| Single medication | 49 (48%) | 26 (39%) | **23  (64%)** |  |
| Multiple medications | 53 (52%) | **40 (61%)** | 13 (36%) |  |
| *^1^* n (%) | | | | |
| *^2^* Pearson’s Chi-squared test or Fisher’s exact test | | | | |

| **Characteristic** | **Overall** N = 110*^1^* | **Treatment group** | | **p-value***^2^* |
| --- | --- | --- | --- | --- |
|  |  | **Surgery** N = 70*^1^* | **Cardiac catheterization** N = 40*^1^* |  |
| Signs/symptoms leading to diagnosis of hsPDA: |  |  |  |  |
| Abnormal pulse | 13 (12%) | 5 (7.1%) | 8 (20%) | 0.064 |
| Hyperactive precordium | 9 (8.2%) | 6 (8.6%) | 3 (7.5%) | >0.9 |
| Arterial hypotension | 12 (11%) | 11 (16%) | 1 (2.5%) | 0.053 |
| Respiratory failure | 60 (55%) | 40 (57%) | 20 (50%) | 0.5 |
| Routine echocardiography | 88 (80%) | 58 (83%) | 30 (75%) | 0.3 |
| Renal insufficiency | 7 (6.4%) | 7 (10%) | 0 (0%) | **0.047** |
| Cardiomegaly | 4 (3.6%) | 4 (5.7%) | 0 (0%) | 0.3 |
| NEC (all stages) | 5 (4.5%) | 3 (4.3%) | 2 (5.0%) | >0.9 |
| Infection/Sepsis | 9 (8.2%) | 9 (13%) | 0 (0%) | **0.025** |
| IVH ≥ grade 2 | 14 (13%) | 9 (13%) | 5 (13%) | >0.9 |
| Pulmonary hemorrhage | 7 (6.4%) | 5 (7.1%) | 2 (5.0%) | >0.9 |
| Heart murmur | 10 (9.1%) | 7 (10%) | 3 (7.5%) | 0.7 |
| Peripheral perfusion | 4 (3.6%) | 1 (1.4%) | 3 (7.5%) | 0.14 |
| PDA: |  |  |  |  |
| PDA diameter (mm) | 2.50 (2.00, 3.00) | 2.30 (2.00, 2.80) | 2.50 (2.00, 3.00) | 0.4 |
| Missing | 20 | 13 | 7 |  |
| Time of diagnosis of hsPDA (day of life) | 6 (4, 12) | 6 (4, 11) | 6 (3, 17) | 0.9 |
| Missing | 3 | 2 | 1 |  |
| V max. (LRS in PDA) | 2.00 (1.50, 2.50) | 2.00 (1.50, 2.40) | 2.00 (1.70, 3.00) | 0.5 |
| Missing | 55 | 36 | 19 |  |
| LA/Ao | 1.93 (1.74, 2.20) | 1.92 (1.79, 2.20) | 1.98 (1.70, 2.00) | 0.8 |
| Missing | 41 | 22 | 19 |  |
| Intervention: |  |  |  |  |
| Weight at surgery/catheter (g) | 995 (843, 1290) | **915** (783, 1110) | 1200 (915, 1950) | **<0.001** |
| Missing | 10 | 10 | 0 |  |
| Age at surgery/catheter (day of life) | 29 (22, 43) | 28 (22, 39) | 30 (21, 66) | 0.2 |
| Missing | 2 | 1 | 1 |  |
| Invasive ventilation before surgery/catheter | 74 (67%) | **57** **(81%)** | 17 (43%) | **<0.001** |
| Non-invasive ventilation before surgery/catheter | 29 (26%) | 14 (20%) | 15 (38%) | 0.045 |
| PDA closed at discharge | 96 (100%) | 60 (100%) | 36 (100%) | >0.9 |
| Missing | 14 | 10 | 4 |  |
| *^1^* Median (Q1, Q3); n (%) | | | | |
| *^2^* Wilcoxon rank sum test; Pearson’s Chi-squared test; Fisher’s exact test | | | | |

| \| **Surgery group** \| **N = 70***^1^* \| \| --- \| --- \| \| Type of closure: \|  \| \| Ligature \| 23 (33%) \| \| Clipping \| 46 (66%) \| \| Other \| 1 (1.4%) \| \| Surgical site: \|  \| \| NICU \| 52 (74%) \| \| Catheter \| 1 (1.4%) \| \| Operating room \| 12 (17%) \| \| External \| 3 (4.3%) \| \| External transfer \| 2 (2.9%) \| \| Surgical team: \|  \| \| Pediatric heart surgery \| 57 (83%) \| \| Heart surgery \| 4 (5.8%) \| \| Pediatric surgery \| 8 (12%) \| \| Missing \| 1 \| |  |
| --- | --- | --- | --- | --- | --- | --- | --- | --- | --- | --- | --- | --- | --- | --- | --- | --- | --- | --- | --- | --- | --- | --- | --- | --- | --- | --- | --- | --- | --- | --- | --- | --- | --- |
| **Surgery group** | **N = 70***^1^* |
| Complications | 10 (15%) |
| Missing | 2 |
| Pneumothorax | 1 (1.4%) |
| Vocal cord palsy | 1 (1.4%) |
| Bleeding | 1 (1.4%) |
| Blood transfusion | 3 (4.3%) |
| Remaining Shunt | 1 (1.4%) |
| *^1^* n (%) | |
|  | |

| **Catheter group** | **N = 40***^1^* |
| --- | --- |
| Device type |  |
| Piccolo Occluder | 21 (62%) |
| Vascular Plug | 7 (21%) |
| Coil | 3 (8.8%) |
| Other/not specified | 3 (8.8%) |
| Missing | 6 |
| Site of intervention: |  |
| NICU | 17 (44%) |
| Catheter laboratory | 18 (46%) |
| Operating room | 1 (2.6%) |
| External | 2 (5.1%) |
| External transfer | 1 (2.6%) |
| Missing | 1 |
| Intervention done by: |  |
| Other | 6 (16%) |
| Pediatric cardiology | 32 (84%) |
| Missing | 2 |
| Complications | 10 (26%) |
| Missing | 1 |
| Haematoma | 1 (2.5%) |
| Remaining Shunt | 0 (0%) |
| Thrombosis | 1 (2.5%) |
| Loss of pulse | 2 (5.0%) |
| Blood transfusion | 1 (2.5%) |
| Device dislocation | 1 (2.5%) |
| LPA stenosis | 2 (5.0%) |
| *^1^* n (%) | |
|  | |

| **Outcome** | **Overall** N = 110*^1^* | **Treatment group** | | **p-value***^2^* |
| --- | --- | --- | --- | --- |
|  |  | **Surgery** N = 70*^1^* | **Cardiac catheterization** N = 40*^1^* |  |
| Patients survived | 98 (91%) | 59 (87%) | 39 (98%) | 0.088 |
| Missing | 2 | 2 | 0 |  |
| *^1^* n (%) | | | | |
| *^2^* Fisher’s exact test | | | | |

| **Outcome** | **Overall** N = 110*^1^* | **Treatment group** | | **p-value***^2^* |
| --- | --- | --- | --- | --- |
|  |  | **Surgical** N = 70*^1^* | **Cardiac catheterization** N = 40*^1^* |  |
| IVH | 29 (27%) | 18 (27%) | 11 (28%) | 0.9 |
| Missing | 4 | 3 | 1 |  |
| IVH ≥ grade 2: |  |  |  | 0.7 |
| Grade 2 | 8 (28%) | 6 (33%) | 2 (18%) |  |
| Grade 3 | 9 (31%) | 5 (28%) | 4 (36%) |  |
| Parenchymal lesions | 12 (41%) | 7 (39%) | 5 (45%) |  |
| NEC | 7 (6.7%) | 6 (9.0%) | 1 (2.6%) | 0.4 |
| Missing | 5 | 3 | 2 |  |
| NEC Bell staging: |  |  |  | 0.14 |
| Stage I | 3 (43%) | 3 (50%) | 0 (0%) |  |
| Stage II | 1 (14%) | 0 (0%) | 1 (100%) |  |
| Stage III | 3 (43%) | 3 (50%) | 0 (0%) |  |
| BPD | 70 (69%) | 49 (79%) | 21 (54%) | **0.008** |
| Missing | 9 | 8 | 1 |  |
| BPD grade: |  |  |  | 0.8 |
| Mild | 12 (18%) | 9 (20%) | 3 (16%) |  |
| Moderate | 29 (45%) | 19 (41%) | 10 (53%) |  |
| Severe | 24 (37%) | 18 (39%) | 6 (32%) |  |
| Missing | 5 | 3 | 2 |  |
| ROP | 48 (51%) | 34 (59%) | 14 (39%) | 0.063 |
| Missing | 16 | 12 | 4 |  |
| ROP staging: |  |  |  | >0.9 |
| Stage 1 | 8 (17%) | 6 (19%) | 2 (14%) |  |
| Stage 2 | 15 (33%) | 10 (31%) | 5 (36%) |  |
| Stage 3 | 22 (48%) | 15 (47%) | 7 (50%) |  |
| Stage 4 | 0 (0%) | 0 (0%) | 0 (0%) |  |
| Stage 5 | 1 (2.2%) | 1 (3.1%) | 0 (0%) |  |
| Missing | 2 | 2 | 0 |  |
| *^1^* n (%) | | | | |
| *^2^* Pearson’s Chi-squared test | | | | |
