# Supplementary figures and images for: Frequency and outcomes of surgical and transcatheter closure of patent ductus arteriosus in preterm infants in Germany—a prospective nationwide hospital-based surveillance study
Source: Eur J Pediatr. 2026 May 20;185(6):416. doi: 10.1007/s00431-026-07073-4 (PMC13186873; doi:10.1007/s00431-026-07073-4)

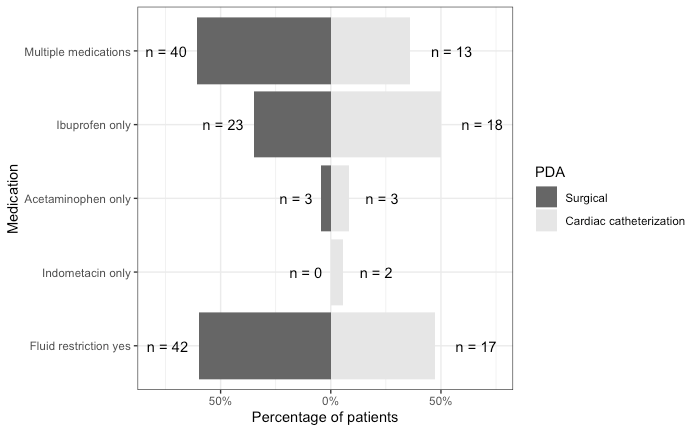

Supplement: Supplementary file 2 — Supplementary Fig. 1: Rate of preterm infants in (%) of the surgical or catheterization group receiving pharmacotherapy with indomethacin, paracetamol, or ibuprofen (multiple therapy possible) with the total number of patients of each group beside the pillars. (PNG 25.0 KB) [file 431_2026_7073_MOESM2_ESM.png]
